# Impact of a Multimodal Improvement Strategy to Promote Hand Hygiene at a Hospital in Mauritius

**DOI:** 10.7759/cureus.15812

**Published:** 2021-06-21

**Authors:** Dooshanveer C Nuckchady

**Affiliations:** 1 Infectious Diseases, Victoria Hospital, Quatre-Bornes, MUS

**Keywords:** hand hygiene, compliance, intervention, multimodal, mauritius

## Abstract

Introduction

Limited data is available on which interventions are likely to improve compliance to hand hygiene, especially in underdeveloped countries. The objective of this study is to explore whether the introduction of a particular bundle of strategies to improve hand hygiene is effective.

Material and methods

In this pre-post study, a multimodal strategy comprised of educating healthcare staff, using reminders, providing feedback and increasing the availability of soap and alcohol, was implemented over a period of one year from 2019 to 2020. Trained observers assessed compliance to hand hygiene before and after the intervention.

Results

A total of 143 hand hygiene opportunities were observed. Hand hygiene compliance rate did not improve despite the introduction of multiple measures.

Conclusions

Other approaches should be considered to promote hand hygiene. The choice of which strategies to use should be adapted to the local setting. For instance, in some healthcare facilities, emphasis should be placed on leadership support and on the training of hand hygiene champions.

## Introduction

Compliance rate to hand hygiene (HH) in developing countries is known to be poor. This can be a risk factor for the spread of multi-drug resistant organisms. A study conducted at an intensive care unit in Mauritius found a high prevalence of antibiotic resistance: 86% of *Acinetobacter spp.*, 30% of *Enterobacteriaceae* and 80% of *Pseudomonas spp.* were carbapenem resistant [1]. Since this is considered a serious issue, a non-randomized pre-post study was carried out to investigate whether the implementation of multimodal strategies could help to improve HH. Of note, a Cochrane review has suggested that only weak evidence is available to demonstrate the utility of many interventions in ameliorating HH [2]. Hence, this study adds valuable information to the current literature.

## Materials and methods

In May 2019, healthcare workers (HCW) at a 600-bed tertiary hospital in Mauritius were observed for one month to assess their compliance to HH. The targeted population included physicians, nurses, and healthcare assistants. All hospital departments including the emergency, intensive care, surgical and internal medicine wards, were involved except for operating theaters, laboratories, and administrative units.

From July 2019 to July 2020, the following steps were introduced: posters on how to perform HH were displayed at each sink, several educational sessions were organized by nursing officers and doctors, alcohol dispensers were made more accessible, feedback regarding the performance on HH was provided and soap was made available in a larger proportion of toilets. Due to the COVID-19 pandemic, hygiene was emphasized throughout 2020. Training consisted of one-hour PowerPoint presentations; at least 20 participants were invited to attend per session.

In October 2020, for a period of one month, direct observation based on the HH steps described by the World Health Organization (WHO) was once again carried out to determine whether any improvement in HH occurred. WHO’s hand hygiene observation tool was filled in by trained staff [3].

A checklist was used to systematically review any barriers that could hinder the implementation of HH. Along with several other items, this checklist assessed the availability of alcohol-based hand rub, the functionality of washbasins, the presence of HH posters and the accessibility of hand towels for single use.

Data were analyzed using IBM SPSS Statistics version 20 and a p-value of less than 0.05 was considered to be statistically significant.

This study was approved by the Ethics Committee of the Ministry of Health and Wellness of Mauritius.

## Results

Before the intervention, out of 43 HH opportunities, staff used alcohol or washed their hands only 5 times, which constituted a compliance rate of 12%. These opportunities included but were not limited to performing a physical examination, taking vital signs, inserting Foley catheters, placing IV cannulas, and taking glucose readings.

After the intervention, out of 100 HH opportunities, HCW were compliant only once (i.e., 1% compliance rate; p-value for the z-test to compare proportions using difference = 0.017).

Of note, during that period, the proportion of alcohol dispensers that were functional increased from 64% to 85%, the number of sinks with a washing agent increased from 34% to 42% and the percentage of toilets with soaps increased from 21% to 29%. Yet, the amount of alcohol used per 1,000 patient days dropped by 18%.

Table 1 summarizes the results of the study and describes the interventions that were instituted.

**Table 1:** This table summarizes the key interventions that were introduced and highlights the outcomes of the study. * HH = Hand Hygiene.

	Pre-intervention	Post-intervention	P-value
Number of HH* opportunities	43	100	--
Nursing officers	13	42	0.18
Doctors	22	33	0.04
Healthcare assistants	8	25	0.41
Type of interventions			
% of functional alcohol dispensers	64%	85%	0.06
% of beds with alcohol dispensers	6.3%	1.3%	0.001
% of sinks with disposable towel	0%	0%	1
% of sinks with washing agents	34%	42%	0.52
% of toilets with soaps	21%	29%	0.56
Training sessions were conducted on HH	No	Yes	--
Posters were placed on HH	No	Yes	--
Outcomes			
Amount of alcohol used in L per 1,000 patient-days	10	8.1	--
HH compliance rate	12%	1%	0.017

## Discussion

This is the first study to report the implementation of a multifaceted hand hygiene improvement strategy in the Mauritian healthcare setting.

HCW were asked to provide an explanation regarding their lack of compliance. Out of the 11 staff that were interviewed, 8 persons (73%) thought that the lack of functional sanitizers at points of care were one of the main reasons for the failure to adhere to HH and 3 persons (27%) believed there was insufficient institutional emphasis, inadequate training and a lack of guidelines on the topic.

During the period of intervention, despite constant advocacy to provide adequate resources to improve HH, the number of alcohol dispensers within 1 meter of a patient bed dropped by 5 times, mostly because the dispensers were preferentially hoarded next to the nursing station. Moreover, disposable paper towel remained unavailable at the washing basins and in toilets. Furthermore, attendance during training sessions was often modest, mostly because of a lack of interest on the topic and a general paucity of awareness on the importance of HH.

The negative results of this study can be explained by a lack of accountability of staff, insufficient oversight by the administration over the issue of hygiene, inadequate hospital leadership, a high ratio of patients to nurses and the absence of a full-time, functional Infection Prevention and Control (IPC) team. In addition, due to the mental toll of the COVID-19 pandemic on the HCW and the absence of local cases of SARS-CoV-2 in the community of Mauritius in October 2020, it is possible that staff let their guard down because of alert fatigue. Moreover, the wearing of gloves could have reduced HH compliance, a behavior that has been reported elsewhere in the literature [4].

Table 2 summarizes the strategies that can be used to improve HH. Implementing a single procedure at a facility is likely to fail; hence, the use of a bundle of techniques should be emphasized. Decision makers at healthcare facilities can pick a set of multimodal strategies from the table based on the specific hurdles being faced at their location. Several institutions have found success in the application of particular tools like the Joint Commission Targeted Solutions tool, WHO's Multimodal Hand Hygiene Improvement Strategy, Continuous Quality Improvement processes and through the use of behavioral change theories like the Behaviour Centered Design and the Health Action Process Approach [5-10].

**Table 2:** List of interventions that may be used to improve compliance to hand hygiene in the healthcare setting

Interventions to improve hand hygiene
Training: Education, problem-based or group-based discussions, simulations and coaching using automated gaming technology
Application of behavioral change theory
Auditing, monitoring and feedback, and applying an accurate and transparent quality assurance. Monitoring can be through direct observation, manually through questionnaires or controlled charts, electronically through camera surveillance or via an automated technique through radiofrequency identification technology
Use of posters and other forms of reminders
Competition e.g., through photo contests or regular display of compliance data
Strong leadership, shared accountability, frontline ownership and frequent goal setting
Broad vertical and horizontal communication with provision of a safe hospital environment
Increasing the availability of hand hygiene supplies, placing dispensers at points of care and proper surveillance of the quantity of products in store
Robust administrative and organizational support
Promotional campaigns
Financial incentives
Coaching handwashing champions and role models
Implementing an institutional operational plan to monitor progress e.g., through the use of a standardized register form of hand hygiene corrective actions

This is not the first study to demonstrate a drop in IPC standards during the COVID-19 pandemic. Hess et al. reported a decrease in the use of HH monitoring badges from 2019 to 2020 while Patel et al. described an increase in the standardized infection ratio of central line-associated bloodstream infections by 28% when comparing data of 2020 to those of 2019 [11,12]. In France, the HH rate dropped by 9.87% post-lockdown after the first wave of COVID-19; the authors explained that the behavior of HCW was probably altered based on their perceived risk of catching COVID-19 i.e., compliance to HH correlated with the epidemic curve of SARS-CoV-2 [13]. Moore et al. also noted a fall in the adherence to HH from 60% to 54% as the pandemic of COVID-19 progressed [14].

The spread of multi-drug resistant organisms within certain hospitals in Mauritius can be explained by poor HH. Also, the elevated rate of COVID-19 among HCW (especially during the second wave that started in March 2021) may be linked to deficient IPC standards [15]. Furthermore, additional failings in IPC are likely to play a role in the high prevalence of antibiotic resistance among microbes in the country. For instance, according to an audit in 2020, only 25% of patients harboring multi-drug resistant organisms were isolated and the use of barrier precautions was often scanty [16].

One main advantage of this study is the long duration of the study of over a year which aimed at assessing the sustainability of the interventions. In addition to this, it is easily reproducible in most settings. Several studies examined the effects of these strategies over a period of only a few weeks but this fails to account for long term effects. The Hawthorne bias was unlikely to be present given that HCW were unaware when they were being observed. The weaknesses include the fact that this study was carried out at a single center, a control group was absent and its sample size was small. However, the author believes the negative results of this study can help to inform subsequent strategies to improve HH, not only to reduce the prevalence of drug resistant microbes but also to stop the spread of harmful organisms in the community, especially during outbreaks [17].

## Conclusions

In the future, multimodal strategies to tackle deficiencies in HH should take a different approach from the one taken in this study. For instance, dispensers should be placed at points of care, health behavior change theory should be used to engage staff to help sustain long-term improvement, administrative support must be present and HH champions should be trained. Other strategies that may improve HH and require further investigation include the organization of HH competitions and simulation exercises, and the use of automated electronic hand hygiene compliance monitoring systems. Following this study and through noteworthy support from the WHO, the Ministry of Health and Wellness of Mauritius agreed to set up a National IPC Committee in 2021 in order to further scrutinize IPC practices and to ensure that safe practices are followed judiciously in the country.

## References

[1] Nuckchady DC, Boolaky SH (2020). The prevalence of multi-drug resistant organisms and their outcomes in an ICU in Mauritius: an observational study. Asian J Med Health.

[2] Gould DJ, Moralejo D, Drey N, Chudleigh JH, Taljaard M (2017). Interventions to improve hand hygiene compliance in patient care. Cochrane Database Syst Rev.

[3] (2009). World Health Organization. Observation Form. https://www.who.int/gpsc/5may/Observation_Form.doc.

[4] Fuller C, Savage J, Besser S, Hayward A, Cookson B, Cooper B, Stone S (2011). "The dirty hand in the latex glove": a study of hand hygiene compliance when gloves are worn. Infect Control Hosp Epidemiol.

[5] Anderson R, Rosenberg A, Garg S (2021). Establishing the Foundation to Support Health System Quality Improvement: using a hand hygiene initiative to define the process. J Patient Saf.

[6] Farhoudi F, Sanaei Dashti A, Hoshangi Davani M, Ghalebi N, Sajadi G, Taghizadeh R (2016). Impact of WHO Hand Hygiene Improvement Program implementation: A quasi-experimental trial. Biomed Res Int.

[7] Mestre G, Berbel C, Tortajada P (2012). "The 3/3 strategy": a successful multifaceted hospital wide hand hygiene intervention based on WHO and continuous quality improvement methodology. PLoS One.

[8] Sands M, Aunger R (2021). Development of a behaviour change intervention using a theory-based approach, Behaviour Centred Design, to increase nurses' hand hygiene compliance in the US hospitals. Implement Sci Commun.

[9] von Lengerke T, Lutze B, Krauth C, Lange K, Stahmeyer JT, Chaberny IF (2017). Promoting hand hygiene compliance: PSYGIENE—a cluster-randomized controlled trial of tailored interventions. Dtsch Arztebl Int.

[10] Ben Fredj S, Ben Cheikh A, Bhiri S (2020). Multimodal intervention program to improve hand hygiene compliance: effectiveness and challenges. J Egypt Public Health Assoc.

[11] Hess OCR, Armstrong-Novak JD, Doll M (2020). The impact of coronavirus disease 2019 (COVID-19) on provider use of electronic hand hygiene monitoring technology. Infect Control Hosp Epidemiol.

[12] Patel PR, Weiner-Lastinger LM, Dudeck MA (2021). Impact of COVID-19 pandemic on central-line-associated bloodstream infections during the early months of 2020, National Healthcare Safety Network. Infect Control Hosp Epidemiol.

[13] Huang F, Armando M, Dufau S, Florea O, Brouqui P, Boudjema S (2021). COVID-19 outbreak and healthcare worker behavioural change toward hand hygiene practices. J Hosp Infect.

[14] Moore LD, Robbins G, Quinn J, Arbogast JW (2021). The impact of COVID-19 pandemic on hand hygiene performance in hospitals. Am J Infect Control.

[15] (2021). COVID- 19: Strengthening Infection Prevention and Control Capacity in Mauritius. https://www.afro.who.int/news/covid-19-strengthening-infection-prevention-and-control-capacity-mauritius.

[16] Nuckchady DC (2020). Infection prevention and control: situation analysis at Dr. A. G. Jeetoo Hospital, Mauritius - 2019-2020.

[17] Bloomfield SF, Aiello AE, Cookson B, O'Boyle C, Larson EL (2007). The effectiveness of hand hygiene procedures in reducing the risks of infections in home and community settings including handwashing and alcohol-based hand sanitizers. Am J Infect Control.

